# Quorum Sensing in ESKAPE Bugs: A Target for Combating Antimicrobial Resistance and Bacterial Virulence

**DOI:** 10.3390/biology11101466

**Published:** 2022-10-06

**Authors:** Sirijan Santajit, Nitat Sookrung, Nitaya Indrawattana

**Affiliations:** 1 Department of Medical Technology, School of Allied Health Sciences, Walailak University, Nakhon Si Thammarat 80160, Thailand; 2 Research Center in Tropical Pathobiology, Walailak University, Nakhon Si Thammarat 80160, Thailand; 3 Department of Microbiology and Immunology, Faculty of Tropical Medicine, Mahidol University, Bangkok 10400, Thailand; 4 Biomedical Research Incubator Unit, Department of Research, Faculty of Medicine Siriraj Hospital, Mahidol University, Bangkok 10700, Thailand

**Keywords:** antibiotic resistance, acyl homoserine lactones, anti-QS, biofilm, ESKAPE, quorum sensing, multidrug resistance, virulence factors

## Abstract

**Simple Summary:**

Quorum sensing in ESKAPE (*Enterococcus faecium*, *Staphylococcus aureus*, *Klebsiella pneumoniae*, *Acinetobacter baumannii*, *Pseudomonas aeruginosa*, and *Enterobacter* spp.) bacteria denotes a global threat to public health. The acquisition of antimicrobial resistance genes, virulence production, and biofilm formation by ESKAPE pathogens has reduced the treatment options for serious infections. QS has been well recognized as being involved in the pathogenesis and antibiotic resistance. More understanding of QS mechanistic would also aid in the prediction of underlying or even unknown mechanisms of antimicrobial resistance and bacterial pathogenesis. In this review, we describe the known antibiotic resistance and pathogenesis caused by QS as well as the strategies to control QS in these pathogens.

**Abstract:**

A clique of *Enterococcus faecium*, *Staphylococcus aureus*, *Klebsiella pneumoniae*, *Acinetobacter baumannii*, *Pseudomonas aeruginosa*, and *Enterobacter* spp. (ESKAPE) bugs is the utmost causative agent responsible for multidrug resistance in hospital settings. These microorganisms employ a type of cell–cell communication termed ‘quorum sensing (QS) system’ to mediate population density and synchronously control the genes that modulate drug resistance and pathogenic behaviors. In this article, we focused on the present understanding of the prevailing QS system in ESKAPE pathogens. Basically, the QS component consisted of an autoinducer synthase, a ligand (e.g., acyl homoserine lactones/peptide hormones), and a transcriptional regulator. QS mediated expression of the bacterial capsule, iron acquisition, adherence factors, synthesis of lipopolysaccharide, poly-*N*-acetylglucosamine (PNAG) biosynthesis, motility, as well as biofilm development allow bacteria to promote an antimicrobial-resistant population that can escape the action of traditional drugs and endorse a divergent virulence production. The increasing prevalence of these harmful threats to infection control, as well as the urgent need for effective antimicrobial strategies to combat them, serve to highlight the important anti-QS strategies developed to address the difficulty of treating microorganisms.

## 1. Introduction

Bacterial quorum sensing (QS) is the intercellular communication system among microorganisms. This phenomenon is mediated by their specific hormone-like signaling molecules called ‘auto-inducers’, which interact with their cognate receptors, thus allowing bacteria to sense the population densities, orchestrate gene expression, and regulate several physiological activities, such as the expression of antibiotic resistance, virulence determinants, motility, conjugation, plasmid transfer, biofilm formation, and interactions with eukaryotic host cells [1,2,3,4]. Recently, ESKAPE organisms, which encompass six leading causative agents of nosocomial infections (*Enterococcus faecium*, *Staphylococcus aureus*, *Klebsiella pneumoniae*, *Acinetobacter baumannii*, *Pseudomonas aeruginosa*, and *Enterobacter* spp.), were shown to be capable of ‘escaping’ conventional antimicrobial treatments, thus evolving from microorganisms with multidrug resistance to those with pan-drug resistance and becoming ‘superbugs’ [5].

Virulence factors are the molecules that assist the bacterium in colonizing the host and initiating diseases. Several virulence determinants are involved in the step of attachment, such as pili, fimbria, fibronectin-binding proteins, PNAG, and adhesins. Additionally, in the step of invasion and tissue damage, many exoenzymes and toxins interplay in this mechanism, including glycohydrolases (hyaluronidase), nucleases (DNase), phospholipases (phospholipase C), proteases (collagenase, gelatinase, elastase, oligopeptide permease), lipid A, cytolysins, exotoxins, alpha-toxins, toxic shock syndrome toxin, hemolysins, leucocidins, coagulase, and pigments, etc. [1,2,3,4]. Antibiotic resistance among these pathogens has been considered a major public health concern worldwide [6,7]. The drug resistance mechanisms in ESKAPE organisms include target alteration, enzyme inactivation, porin loss, efflux pump, and biofilm formation [8]. Several studies have found that the QS system regulates antimicrobial resistance and pathogenicity in these pathogens [9]. Generally, the expression of drug resistance phenotypes through the QS system occurs according to the following key steps: synthesis of QS signaling molecules, release of signal ligands to the milieu, sensing of the signal molecules at a high level of cell density, binding to the transcriptional regulator or R-protein, transporting the ligand–receptor complex from the cell, binding of the complex to the promoter region of the target gene, and transcription of drug resistance-associated genes, leading to phenotypic manifestation [10].

**Figure 1:**
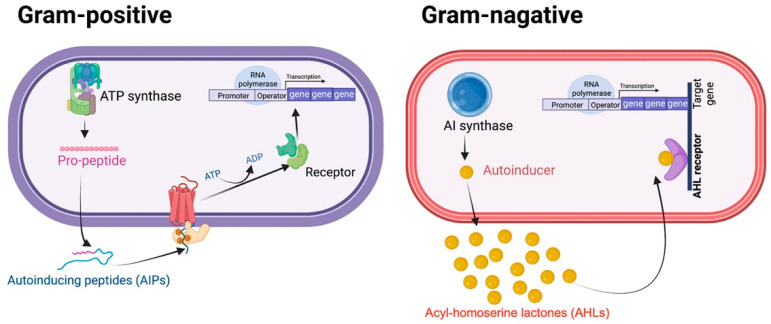
Schematic illustration of quorum sensing systems in bacteria. In Gram-positive bacteria, AIPs engage the receptor and induce the regulator for target gene expression. AIP synthase functions similarly to AI synthase in Gram-negative bacteria. In Gram-negative bacteria, the AHL receptor (LuxR) senses the signaling AHL molecules outside of the cell and forms a contact interaction with them. The AI synthase participates in the synthesis of these signal molecules. When necessary, LuxI (referring to AHL synthase) suppresses the signal. The AHL-receptor complex interacts with the regulator and contributes to the regulation of the target gene. AIP, or auto-inducing peptide; AHL, acyl homoserine lactone.

In general, Gram-positive and Gram-negative bacteria use QS to control a diverse array of vital bacterial behaviors and biological characteristics. The QS circuit consists of main components, including autoinducer synthases, signaling ligands (autoinducers or self-inducers), transcriptional regulators, and target genes (Figure 1). Gram-positive bacteria establish oligopeptides, so-called ‘autoinducing peptides’ (AIPs), which are short peptide chains, as the major autoinducers (AIs) [11]. The mature AIPs can interact with the transmembrane histidine kinase receptor, resulting in autophosphorylation of the transcriptional regulator, consequently triggering the expression of the target gene. In contrast, Gram-negative organisms use acylated homoserine lactones as signal molecules [12]. The derivatives of *N*-acyl homoserine lactones (AHLs), which are classified as autoinducers-1 (AI-1), are predominantly renowned for mediating intraspecies communication among Gram-negative bacteria. Typically, AHL molecules diverge in the length of their carbon chains. Short-chain AHLs (with 4–8 carbon atoms) are able to simply diffuse across the bacterial cell membrane, whereas the long-chain AHLs (with 10–14 carbon atoms) require an active transport system to facilitate their transit through the membrane. Moreover, furanosyl borate diester, which is categorized as an autoinducer-2 (AI-2), is found in both Gram-positive and Gram-negative bacteria and is responsible for interspecies coordination among them [13,14]. To date, several QS inhibitors have been developed to block and/or abolish QS signaling and subsequently prevent pathogenic activities and drug resistance phenotypes. This review focuses on the QS-mediated drug resistance in ESKAPE pathogens and the current state of anti-QS approaches as a potential alternative to traditional therapy in the future.

## 2. QS-Mediated Drug Resistance and Bacterial Virulence in ESKAPE Pathogens

Nosocomial ESKAPE organisms employ AI synthases to generate and accumulate QS signal molecules, thus synchronizing their specific receptors. Subsequently, they stimulate the expression of target genes and acquire pathogenic and antimicrobial characteristics. Typically, Gram-positive bacteria utilize small peptide signals for sensing through the two-component histidine kinase system, which is mainly of the RNPP (Rap, NprP, PlcR, and PrgX regulatory proteins) and Rgg (transcriptional regulator of glucosyltransferase) type, whereas most Gram-negative bacteria control QS-related gene expression via the *N*-acyl homoserine lactone (AHL)-mediated LuxR–LuxI homologous system [1,15].

### 2.1. Enterococcus *spp.*

*Enterococcus* spp., such as *E. faecalis* and *E. faecium*, are common infectious pathogens that produce a variety of virulence genes encoding bacteriocins, aggregation factors, and drug resistance determinants [16]. The antibiotic resistance conjugation transfer is mediated by signaling by common QS peptide pheromones functioning as AIs, such as cCF10 (with the sequence ‘LVTLVEV’) produced by PrgQ. The temporary donor cells harbor resistance genes on pCF10, which is the tetracycline resistance conjugative plasmid, in response to the small signal peptides secreted by recipient cells. These AIs can pass through the PrgZ–Opp complex and the ATP-binding cassette transport system, to then accumulate in the extracellular environment and sense the ligand-binding protein, thus upregulating the conjugation genes that trigger conjugation-related mating functions throughout a population. After the bacterial recipients acquire plasmids, they confer the tetracycline resistance phenotype [17,18]. Similarly, the bacteria occupy an octapeptide pheromone known as cAD1 (with the sequence LFSLVLAG) for QS synchronization. Subsequently, the transfer of the mobile elements called pAD1, which encode a cytolysin (hemolysin/bacteriocin) and an aggregation constituent, has been shown to contribute to pathogenicity [19].

**Figure 2:**
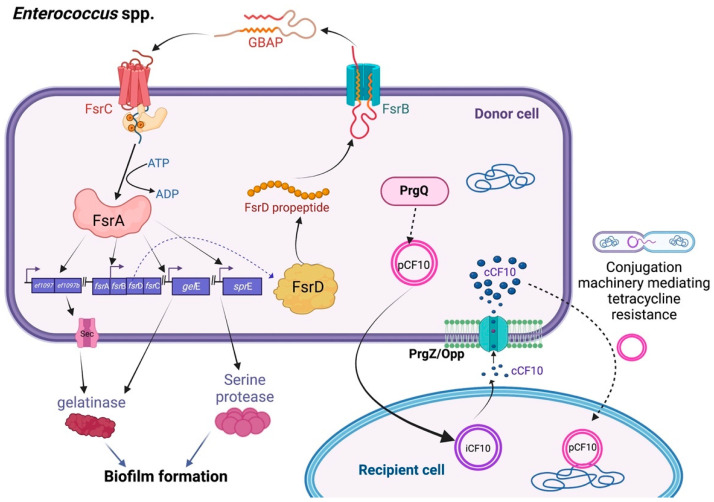
Diagram of the major quorum sensing pathways of *Enterococcus* spp. Conjugative transfer in *Enterococcus* spp. Pheromone signaling occurs between the two cell types and is the mechanism by which the plasmid pCF10 is transferred from donor cells to recipient cells. The chromosomally encoded peptide cCF10, which is secreted by recipient cells and internalized by donor cells, triggers the expression of genes important for the conjugative process. Asc10, also known as “aggregation substance,” is specifically expressed by particular cells and allows a stable interaction between the donor and receivers. The pCF10 plasmid is then transferred from donor cells to recipient cells. The QS system in Fsr and its regulation in *Enterococcus* spp. FsrB exports and processes the FsrD propeptide (encoded by *fsr*D) to create the tiny lactone gelatinase biosynthesis-activating pheromone (GBAP). The intracellular response regulator, FsrA, is phosphorylated in response to extracellular GBAP by FsrC, a component of a two-component regulatory system. FsrA then stimulates the expression of the genes *ef1097*, *ef1097b*, the *fsr* locus, *gel*E (encoding a gelatinase), and *spr*E (encoding a serine protease), which lead to biofilm formation.

Moreover, enterococci, especially *E. faecium*, possess other AIs, including a cyclic peptide molecule called gelatinase, biosynthesis-activating pheromone (GBAP). These signal molecules can cooperate with their transmembrane cognate receptors, FsrC, thus facilitating the QS activity [20]. The faecal streptococci regulator (Fsr) locus is encoded by the fsrA, fsrB, and fsrC genes via GBAP (Figure 2). This controls the expression of gelatinase, biofilm formation, and the production of serine proteases and enterocin O16 (cytolysin). The LuxS/autoinducer-2 (AI-2) system, which is another QS regulatory system, has also been proposed to play a role in the persistence of infections and biofilm development through interspecies communication. However, the details of this mechanism warrant further exploration [21,22].

### 2.2. Staphylococcus aureus

**Figure 3:**
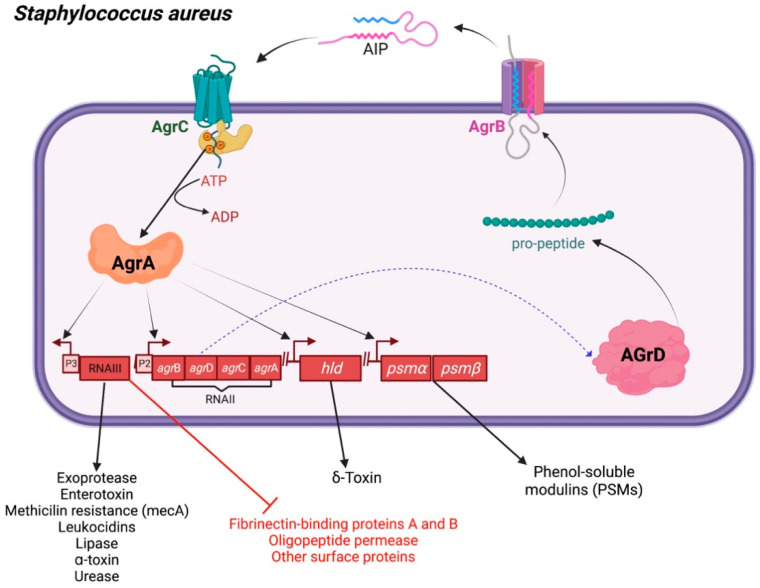
Hierarchical quorum sensing network in *Staphylococcus aureus*. Autoinducing peptide (AIP) synthesis and mechanism of action in *S. aureus*. A two-component signal transduction pathway underlies AIP-mediated quorum sensing in *S. aureus* (AgrC and AgrA). RNAII and RNAIII regions’ P2 and P3 promoters are correspondingly activated by phosphorylated AgrA. AIP is synthesized as a result of RNAII expression, and RNAIII expression controls the synthesis of exoproteins, virulence genes, and δ-hemolysin genes while inhibiting the synthesis of adhesins.

The QS system potentiates *S. aureus* to cause disease according to the expression of various adhesins, toxins, and substances that disrupt the host’s immune system [23]. Most Gram-positive bacteria utilize a two-component QS cascade, which is controlled by an additional gene regulator (*agr*) locus [23]. The agr system comprises RNA, RNAII, and RNAIII transcripts. The RNAII operon comprises *agr* genes, such as *agrB*, *agrD*, *agrC*, and *agr*A. The signaling pathway is initiated by the production of an agrD-encoding peptide, which is then modified by an integral membrane protein named ‘AgrB’. The altered peptide acts as an ultimate AIP [24,25]. This two-component machinery, composed of AgrA and AgrC, as well as of an AIP-binding domain, participates in histidine kinase transduction. Stimulation of the two-component system turns on the RNAII operon, which acts as a regulator of RNAIII transcription [26]. RNAIII can trigger the generation of α-toxin, while inhibiting the expression of the fibronectin-binding proteins A and B, peptide A, oligopeptide permease, coagulase, and other surface proteins (Figure 3).

Bacteria form biofilms as part of their survival mechanisms. The biofilm layer promotes the bacteria’s tolerance to antibiotics and chemical disinfectants. Molecular mechanisms that have been shown to enhance establishment of bacterial biofilm indicate that biofilm virulence factors likely arise through resistance to phagocytosis and other host immune defense mechanisms resulting in persistence in the host niche rendering the chronic infections [3,4,23]. To date, several reports have addressed the regulation of biofilm formation mediated by the LuxS/AI-2 system. In *S. aureus*, the homolog of luxS, which encodes AI-2 synthase, promotes ribosome binding factor (rbf) transcription, consequently increasing the production of polysaccharide intercellular adhesion and adhesion, which positively regulate biofilm formation and have been investigated in other bacteria, e.g., *Escherichia coli*, *Salmonella enterica*, *Klebsiella pneumoniae*, and *Streptococcus pneumoniae* [27].

### 2.3. Klebsiella pneumoniae

*K. pneumoniae* primarily exploits the AI-1 QS system for conducting its cellular processes. Due to the lack of luxI gene homologs for LuxI synthase establishment, this strain encodes a LuxR receptor (acronym, ‘SdiA’), but does not produce AHLs [28,29]. This orphan receptor responds to the exogenous AHL produced by other bacterial strains, thus regulating cell division and the expression of virulence factors, such as fimbriae expression, biofilm formation, and production of QS autoinducers in *K. pneumoniae* [30].

**Figure 4:**
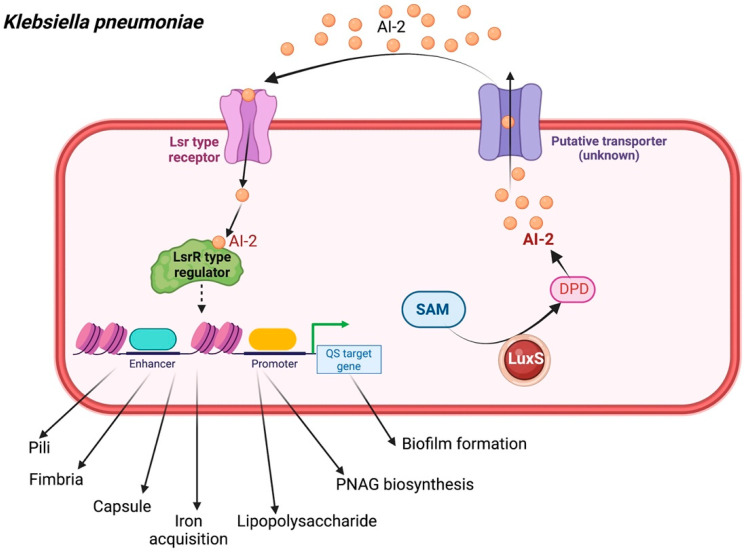
Quorum sensing cascade in *Klebsiella pneumoniae*. AI-2 dependent regulated QS signaling in *K. pneumoniae*. AI-2 is expressed by a *lux*S homolog, as well as *N*-octanoyl homoserine lactone (C8-HSL) and *N*-3-dodecanoyl-L-homoserine lactone (N-3-DL-HSL) (C12-HSL). The *lux*S system is associated with the expression of LPS synthesis-related genes and their expression of capsule, fimbria, pili, LPS, iron acquisition, and PNAG biosynthesis, as well as biofilm formation. SAM, *S*-adenosyl methionine; DPD, 4,5-dihydroxy-2,3-pentanedione.

Similar to other ESKAPE organisms, *K. pneumoniae* also engages in classical QS-mediated bacterial negotiation via the furanosyl borate diester (AI-2) produced by the LuxS synthase and by N-octanoyl homoserine lactone (C8-HSL) and N-3-dodecanoyl-L-homoserine lactone (C12-HSL) [31,32]. When AI-2 is mediated by the LuxS system, the signaling molecule is passed through the transmembrane protein and transferred to the bacterial transporter [33]. Once AI-2 is internalized into the cytoplasm and phosphorylated by LsrK, the phospho-AI-2 further abolishes the LsrR repression of the lipolysis-stimulated lipoprotein receptor (lsr) operon, thus resulting in increased AI-2 uptake. As optimal cell density is reached, the extracellular AI-2 binds to cognate receptors and the signal transduction pathway is activated. The results revealed the expression of the bacterial capsule, iron acquisition, adherence factors, and synthesis of lipopolysaccharide (LPS), which promote the pathogenicity of the agent. Moreover, the study demonstrated the expression of poly-β-1,6-*N*-acetylglucosamine (PNAG) biosynthesis as well as biofilm development, rendering the drug-resistant *K. pneumoniae* (Figure 4) [34,35,36].

### 2.4. Acinetobacter baumannii

**Figure 5:**
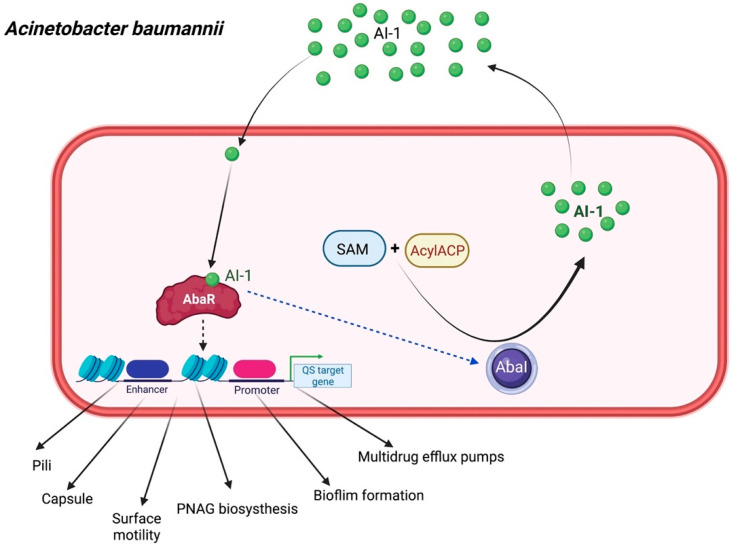
Diagrammatic illustration of quorum sensing signaling in *Acinetobacter baumannii*. AI-1 mediates the QS cascade in *A. baumannii* through the LuxR receptor (AbaR) and LuxI synthase (AbaI) systems. This QS system is crucial for capsule biogenesis, bacterial motility, multidrug efflux pumps, and biofilm development capability. AcylACP, Acyl carrier protein.

The single-complex QS machinery in *A. baumannii* is homologous to the typical LuxI/LuxR system. This machinery mainly comprises AbaI (AI synthase), a signaling AHL molecule, and AbaR (cognate receptor), which is controlled by the abaR/abaI locus [37,38]. When AbaI-generated AHL forms a complex with AbaR, the AbaR–AI complex recognizes a putative lux-box sequence (CTGTAAATTCTTACAG) [39], thus triggering drug resistance, surface motility, and the production of the exopolysaccharide poly-β-1,6-N-acetylglucosamine (PNAG), which is essential for adhesion, aggregation, and biofilm formation (Figure 5) [40]. This organism creates medium-to-long-chain AHLs with their acyl side chains, ranging from C6 to C8 and C10 to C16, such as unsubstituted C10-HSL, C12-HSL, 3-hydroxy-C10-HSL, 3-hydroxy-C12-HSL, unsaturated 3-oxo-C11-HSL, and C14-HSL [41,42]. The most abundant AHL is *N*-(3-hydroxydodecanoyl)-L-homoserine lactone (3-hydroxy-C12-HSL) [37]. Moreover, the loci located between *aba*R and *aba*I encompass a third gene, namely *aba*M, which controls the expression of an uncharacterized RsaM protein family. Previous evidence showed that AbaM downregulates AHL synthesis in *A. baumannii* and modulates surface motility and biofilm formation [43]. Moreover, it regulates *N*-acyl homoserine lactone (AHL)-dependent QS in other beta- and gamma-proteobacteria, such as *Burkholderia* spp., *Halothiobacillus neapolitanus*, and *Pseudomonas fuscovaginae* [44].

Furthermore, many studies have reported the role of the efflux system in the resistance–nodulation–cell division (RND) systems, AdeABC, AdeIJK, and AdeFGH, which share structural similarity with the MexAB pumps of *P. aeruginosa*. This machinery is used for the transport of QS molecules, virulence factors, and antibiotics in *A. baumannii* [45,46]. This finding indicated the correlation between efflux-pump-mediated QS and antibiotic resistance in this organism. Basically, iron is required for many physiological activities in pathogens, including DNA replication, transcription, metabolism, and energy generation via respiration. Some reports demonstrated that restricted concentrations of iron can positively regulate QS signaling molecules, thus increasing population persistence and virulence production in the bacteria [38,47,48].

### 2.5. Pseudomonas aeruginosa

The eradication of *P. aeruginosa* is frequently challenging because it is resistant to several antibiotics and generates various virulence determinants. The ability of *P. aeruginosa* to develop a biofilm is another important virulence trait that hampers its removal. Similar to other ESKAPE bacteria, *P. aeruginosa* uses a small diffusible signal molecule to mediate QS. The predominant QS signals produced by this organism are *N*-butanoyl-L-homoserine lactone (C4-HSL), 3-oxo-dodecanoyl-L-homoserine lactone (3-oxo-C12-HSL), and 2-heptyl-3-hydroxy-4-quinolone (PQS; *Pseudomonas* quinolone signal) [28,49,50].

The LasI/LasR and RhlI/RhlR systems drive the production of various virulence elements (such as elastase, pyocyanin pigment, and rhamnolipid biosynthesis) and biofilm development, which triggers the reduction of conventional antibiotic susceptibility [51]. These signal ligands, such as 3-oxo-C12-HSL and C4-HSL, are generated by AI synthases, including LasI and RhlI, respectively. Their cognate receptor proteins are LasR and QscR for 3-oxo-C12-HSL, and RhlR for C4-HSL [52]. In addition, the C4-HSL of *P. aeruginosa* expands the expression of the MexAB–OprM efflux pump, which confers resistance to traditional beta-lactam drugs [53].

**Figure 6:**
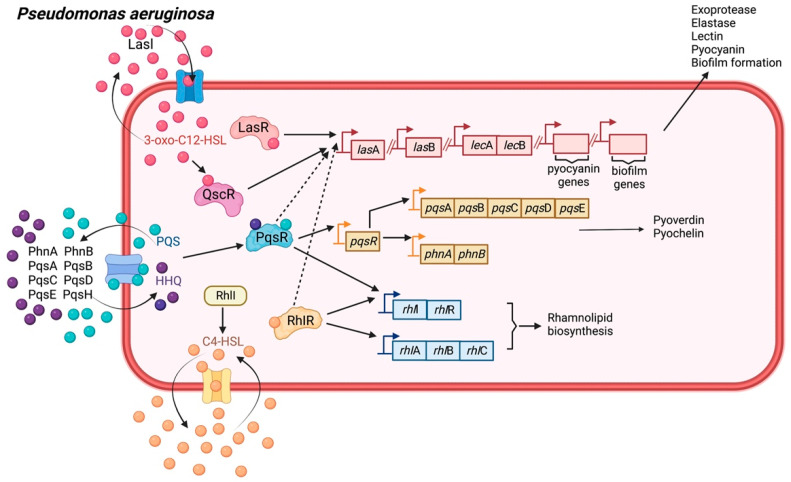
Quorum sensing machinery in *Pseudomonas aeruginosa*. The hierarchical organization of the three major QS systems in *P. aeruginosa* has been recognized as Las, Rhl, and PQS (such as *Pseudomonas* Quinolone Signal). These systems involve the signal synthases LasI, RhlI, PqsABCDEH, and the receptors LasR, RhlR, and PqsR, respectively. Three autoinducer signaling molecules are employed by these systems. The Las and Rhl systems employ two acyl-homoserine lactones (AHL), 3-oxo-C12 HSL, and C4 HSL, whereas PQS is based on 2-alkyl-4-quinolones (i.e., PQS and HHQ). Besides, the cognate receptors for 3-oxo-C12-HSL are also QscR. All three of these systems are interconnected, and Las is regarded as the global activator. The Las system controls exoprotease, elastase, lectin, pyocyanin synthesis, and biofilm development. The Rhl system triggers rhamnolipid biogenesis. The Pqs system positively regulates the Las and Rhl systems and triggers pyoverdine and pyochelin production. Solid arrows indicate direct control of genotypic or phenotypic signal regulation, while dashed arrows indicate additional or alternative transcriptional control for gene expression.

Another QS system that is present in this organism arbitrates a second class of QS signals, including 4-hydroxy-2-alkylquinolines (HAQs) (such as 4-hydroxy-2-heptylquinoline (HHQ) derivatives and 2-heptyl-3,4-dihydroxyquinoline, which is the corresponding dihydroxylated derivative. The HAQ biosynthesis in this pathogen requires a set of genes encoded by the pqsABCDE and phnAB operons. PQS is synthesized by hydroxylation of HHQ by a putative monoxygenase known as PqsH. Both HHQ and PQS act as co-inducing ligands of PqsR, also called multiple virulence factor regulator (MvfR). These activated the QS via their cognate receptor, PqsR [28,52]. Furthermore, PQS plays a role during *P. aeruginosa* infection by deliberating the expression of virulence factors (for instance, pyocyanin, elastase, lectin, and rhamnolipids; and biofilm development) and provoking the inflammation caused by host immune responses (Figure 6) [54].

### 2.6. Enterobacter *spp.*

*Enterobacter* QS signaling, especially in *E. aerogenes* and *E. cloacae*, occupies AI-1, AI-2, and AI-3 as the functional signaling ligands [55]. Although little information related to QS-controlled drug resistance and pathogenesis is available in this group of bacteria, several research efforts have been made to elucidate the complicated mechanisms that contribute to the pathogenicity and antimicrobial resistance of diverse *Enterobacter* genera.

Some evidence has indicated that *Enterobacter* spp. use C4-HSL and C6-HSLs as QS signals [56]. These are generated by a LuxR homolog, which has been found to decrease bacterial adhesion and downregulate biofilm development [57]. In *E. asburiae*, the QS pathway is triggered by C4-HSL and C6-HSL, which bind to their cognate EasR receptor protein (the transcriptional regulator *lux*R homolog), thus triggering QS-associated gene transcription, as well as its related phenotypes and biofilm formation [58].

**Figure 7:**
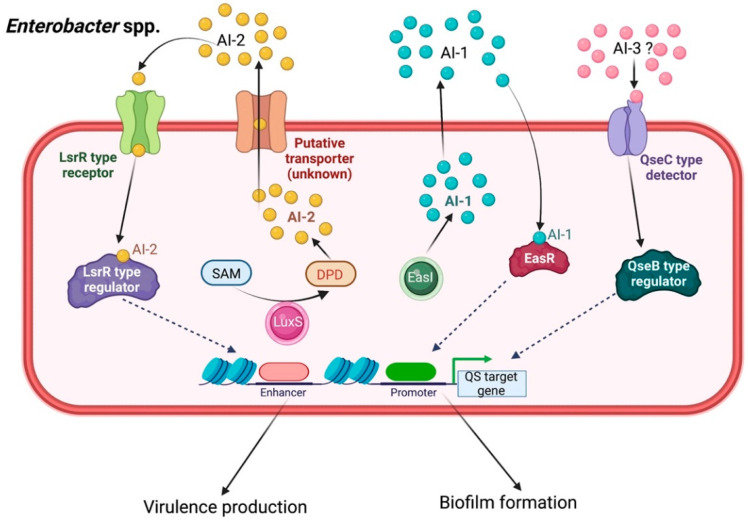
Quorum sensing signaling in *Enterobacter* spp. Three main QS systems play a role in intercellular communication signaling within *Enterobacter* spp. mediated through AI-1, AI-2, and AI-3 cascades to induce virulence expression and biofilm formation.

Intercellular negotiation among *Enterobacter* spp. also occurs via the AI-2-mediated QS system, as the cognate Lsr-type receptors have been found in strains of *E. cloacae*, *E. cancerogenus*, and *E. mori* [59,60]. Mostly in Enterobacteriaceae, such as *E. cloacae* and the enterohemorrhagic *Escherichia coli* O157:H7, the AI-3, epinephrine and norepinephrine, was also reported to modulate QS phenotypes, such as biofilm formation, which is accelerated by the QseC/QseB system (Figure 7) [61].

## 3. Therapeutic Approaches Targeting QS Systems Counteract Drug Resistance and Virulence in ESKAPE Bugs

Disrupting bacterial QS pathways in ESKAPE bacteria seems to be an attractive broad-spectrum remedial strategy for reducing resistance to antimicrobial agents, silencing bacterial pathogenesis, and promoting susceptibility to host immune defenses without eliciting any evolutionary pressure. To date, several protocols have been proposed as potential anti-QS approaches, such as blocking AI synthases, degrading the AIs, inactivating transcriptional regulators, interfering with the ligand–receptor complex, and incorporation with traditional drugs (Table 1) [62,63].

### 3.1. Targeting AI Synthase

Several studies have demonstrated that the inhibition of QS signal synthesis can interrupt the initial step of the QS network, which attenuates AHL-mediated virulence and drug resistance phenotypes. In this manner, several studies have reported the use of natural and synthetic compounds that are structural analogues of the substrates for the AHL synthases *S*-adenosyl methionine (SAM) and acyl-carrier protein (ACP). In *P. aeruginosa*, sinefungin, butyryl-SAM, and L/D-*S*-adenosylhomocysteine can attenuate QS-mediated virulence factors and prevent bacterial infection by inhibiting the secretion of AHLs [64,65].

Evidently, triclosan can reduce the production of AHLs by diminishing the production of enoyl-ACP reductase precursors (FabI) in *S. aureus* [66].

### 3.2. Sequestration of QS Ligands

The degradation of QS signal molecules in Gram-negative bacteria triggers the inactivation of AHLs, which is mediated by enzymatic activity. The major enzymes identified in many *P. aeruginosa* research studies include AHL lactonases, acylases, oxidoreductases, and 3-hydroxy-2-methyl-4(1H)-quinolone 2, 4-dioxygenase. AHL lactonases and AHL acylases function by cutting the amide linkage with different lengths of side chains of AHLs and destroying the lactone ring. AHL lactonases, such as lactonase SsoPox, lactonase Aii810, quorum quenching lactonase enzyme AHL-1 (a novel lactonase cloned by bpiB01 and bpiB04), and lactonase AiiK, are found to reduce the production of extracellular proteases and pyocyanin, rhamnolipids, swarming motility, and biofilm formation, and to prevent bacterial infection [67,68,69,70,71,72]. Furthermore, AHL lactonases have been shown to increase bacterial sensitivity to traditional drugs without affecting bacterial metabolic growth [108]. In *A. baumannii*, the engineered AHL lactonase was also reported to sequester AHL and reduce the *A. baumannii* biomass-associated biofilms, which increases bacterial sensitivity to antibiotics without affecting the growth of *A. baumannii*. Many current agents are intended to directly kill pathogenic bacteria by damaging cell membranes or interfering with fundamental protein synthesis. The widespread use of agents has resulted in major microbial resistance problems, and this selection pressure encourages the evolution of microbial resistance. AHL lactonase has less effect on organism development, implying less selection pressure to drive microorganism evolution. This indicates that anti-QS compounds may be used as potential alternatives to traditional medications [109].

Acylases constitute another enzyme type that can block the QS pathway by hydrolyzing the amide bond of AHLs. Previous research found that *Aspergillus melleus* acylase can degrade C4-LHL, C6-LHL, and 3-oxo-C12-LHL, resulting in decreased pyocyanin synthesis and biofilm formation [73]. Acylase (EC.3.5.1.14) can inactivate AHL inducers, resulting in decreased biofilm biomass [74]. *N*-acyl homoserine lactone acylase PA2385 can destroy the 3-oxo-C12-HSL and 2-heptyl-3-hydroxy-4 (1H)-quinolone, which lessens elastase and pyocyanin biosynthesis [75].

Regarding oxidoreductases, another enzyme can change the acyl side-chain structure of AHLs, thus interfering with the expression of QS signaling. The BpiB09 oxidoreductase was reported to inhibit the activation of 3-oxo-C12-HSL, causing a reduction in bacterial motility, biofilm formation, and pyocyanin production in *P. aeruginosa* [76]. Similarly, oxidoreductases immobilized on a glass surface can inhibit bacterial biofilm development and decrease the growth rate in *K. pneumoniae* [77].

The 3-hydroxy-2-methyl-4 (1H)-quinolone can catalyze the conversion of PQS to *N*-octanoylanthranilic acid and carbon monoxide, thereby downregulating lectin A, pyocyanin, and rhamnolipid [78]. Dioxygenase has been shown to block the quinolone-mediated QS signals via the degradation of 2-heptyl-3-hydroxy-4 (1H)-quinolone of *P. aeruginosa*, thus decreasing the generation of pyocyanin, rhamnolipid, and lectin A [78,110].

Another intriguing anti-QS platform arbitrated by antibodies that target the QS signal molecules has also been reported, such as the RS2-1G9QQ antibody, which prevented the stimulation of the mitogen-activated protein kinase p38 and protected murine bone-marrow-derived macrophages from cytotoxic effects [79]. The XYD-11G2 antibody hydrolyses 3-oxo-C12-HSL, leading to the suppression of bacterial QS signals [80]. Moreover, the engineered human single-chain variable fragments inhibit *P. aeruginosa* 3-oxo-C12-HSL and prevent mammalian cell apoptosis [81].

A previous study of Gram-positive bacteria, including *S. aureus*, demonstrated that the AP4-24H11 antibody targets autoinducing peptide-4, elicits protective activities *in vivo* by attenuating pathogenicity in *S. aureus*-generated-abscess formation in a mouse model, and offers a complete defense against a lethal *S. aureus* challenge [82]. Furthermore, *in vivo* studies have demonstrated the inhibitory activity of synthetic RIP (the amide form of the originally isolated one) by reducing *S. aureus* infections, such as cellulitis, septic arthritis, keratitis, osteomyelitis, and mastitis [83]. The synergistic activity exerted through the combination of synthetic RIP and antibiotics has been reported to act against *S. aureus* biofilms [84].

### 3.3. Blocking of QS Transcriptional Regulators

Inactivation of receptors in QS signaling is an effective strategy for deactivating bacterial virulence and infection. Many studies have revealed that, in *P. aeruginosa*, flavonoids can target the allosteric inhibition of AI-binding receptors, including LasR and RhlR, which affects the transcription of QS-controlled target promoters and suppresses virulence factor production [111]. *N*-decanoyl-L-homoserine benzyl ester can activate a QS control repressor, therefore attenuating protease and elastase activities, swarming motility, and biofilm development [85]. LasR and RhlR can be inhibited by meta-bromo-thiolactone, thus disarming the production of pyocyanin and biofilm formation [86]. The AHL ligands include A4, 4-bromophenyl-PHL B7, 4-iodo PHL C10, and 3-nitro PHL C14, which bind to TraR, LasR, and LuxR, thus inhibiting the production of virulence factors [87].

Moreover, several researchers have reported the effectiveness of virstatin, which is a tiny organic compound, as an inhibitor that prevents *A. baumannii* from expressing anoR, a LuxR-type regulator, which is a homologue of the AbaI/AbaR regulatory system. The effectiveness of virstatin as a T4P pili system biogenesis inhibitor in preventing bacterial movement and initiating biofilm formation has been demonstrated [88].

### 3.4. Alternative Approach for Inhibiting QS Using Probiotics

The treatment or primary prevention of bacterial infections using probiotics has been demonstrated to be successful. However, the effectiveness of these agents has a strain- and disease-specific nature. The benefit of using probiotics as therapeutic agents is that these living microbes frequently have multiple modes of action, such as expressing proteases that specifically destroy Toxin A, inference with toxin attachment sites, immune regulation, and other mechanisms that may include inhibition of QS systems [112].

Studies have shown that certain probiotic strains may interfere with the QS system of ESKAPE bacteria. Another study has shown that *Lactobacillus plantarum* PA100 can prevent the induction of *P. aeruginosa* virulence factors by targeting AHL. According to this investigation, the development of biofilm, elastase, and AHL could be inhibited by the acid filtrate and the neutralized filtrate of *L. plantarum* PA100 [89]. Moreover, C4-HSL and 3-oxo-C12-HSL of *P. aeruginosa* can be destroyed by cell extracts of *L. crustorum* ZHG 2-1, which has been reclassified as *Companilactobacillus crustorum*. Thus, suppression of biofilm formation, loss of swarming and swimming motilities, and reduction of virulence factors (chitinase and protease) were also noted, without altering bacterial growth [90]. In addition, the metabolites of lactic acid bacteria, such as *L. lactis* NCDC 309, *L. rhamnosus* MTCC 5897, *L. rhamnosus* MTCC 5857, *L. fermentum* MTCC 5898, *L. acidophilus* NCDC 15, *L. delbrueckii* subsp. *lactis*, and *L. plantarum* NCDC 372, were found to effectively hinder elastase and biofilm production, as well as *las*I and *rhl*I gene expression in *P. aeruginosa*. It is interesting to note that these supernatants efficiently lower AHL synthesis [91].

In Gram-positive bacteria, the *L. reuteri* RC-14 strain, which acts as a probiotic and is used to treat toxic shock syndrome, creates the small molecules cyclo (L-Phe-L-Pho) and cyclo (L-Tyr-L-Pro), thus disrupting the QS system of toxic strains of *S. aureus*. The TSST-1 gene, which encodes the toxin linked to toxic shock syndrome, was suppressed by this interference [91]. The biosurfactants generated by *L. plantarum* and *P. acidilactici* decrease the expression of AI-2 in a dose-dependent manner, as well as the *cid*A, *ica*A, *dlt*B, *agr*A, *sortase*A, and *sar*A genes, which are related to biofilm development in *S. aureus* [93].

### 3.5. Alternative Approach for Inhibiting QS Using Plant Extracts

Currently, various plant extracts, including the previously mentioned flavonoids and phenolic acids, display potent action against ESKAPE QS [94]. In addition, the previous study showed that eugenol suppressed the synthesis of virulence factors such as elastase, pyocyanin, and biofilm formation in *P. aeruginosa* via the las and pqs QS systems [95,96,97]. Eugenol also prevented the formation of biofilms and the expression of QS synthase genes, particularly *las*I, *rhl*I, and the *rhl*A gene [97]. In methicillin-resistant *S. aureus*, this compound also limits the protease enzymes and pigment production [98]. Another study found that carvacrol (2-methyl-5-(1-methylethyl)-phenol) was effective against biogenesis and QS which lessens the pathogenicity of *P. aeruginosa* by blocking *las*I expression and reducing *las*R expression, as well as biofilm growth and surface motility [99,100].

A diterpene known as phytol has shown anti-QS activity. In this manner, this substance suppresses flagella mobilization, restricts the formation of pyocyanin, and inhibits the establishment of the biofilm in *P. aeruginosa* PAO1 [102,103]. Another terpene with anti-QS properties is sesquiterpene lactones. This compound diminished the QS mediators in *P. aeruginosa* ATCC 27,853 [104]. Similar to the previous example, oleanolic aldehyde coumarate showed inhibitory actions against *P. aeruginosa* biofilm by downregulation of *las*I/*las*R, *rhl*I/*rhl*R, and *gac*A expression [105]. Additionally, other terpenoids, including linalool, hindered the generation of *A. baumannii* biofilms and altered this strain’s surface adhesion. This trait is associated with linalool’s interference with the QS system [106,107].

Several studies have looked into the anti-QS properties of quercetin and its derivatives [113,114,115,116]. The substance has antibiofilm properties against *P. aeruginosa* strain PAO1 and inhibits the synthesis of virulence factors such as pyocyanin, protease, and elastase by lowering *las*I, *las*R, *rhl*I, and *rhl*R gene expression levels.

## 4. Clinical Applications and Future Perspectives

Many anti-QS compounds have been suggested by various researchers to control pathogenesis, infection, and antibiotic resistance in pathogenic organisms, including ESKAPE. Nevertheless, the tolerance, effectiveness, and safety of therapeutic regimens for clinical usage should be investigated. In recent years, the pyrimidine analog 5-fluorouracil (5-FU) is a potent quorum-quencher, inhibiting AI-2 production of MRSA, *Staphylococcus epidermidis*, *E. coli*, and *Vibrio harveyi*, and has gained popularity as an antimetabolite used both topically and systemically for the treatment of actinic keratoses and neoplastic disorders [114]. The anti-QS compounds showed growth inhibition against both Gram-positive and Gram-negative bacteria, and the 5-FU was utilized as an antimetabolite by coating central venous catheters to prevent colonization or infection of a patient’s implanted medical device [115]. When utilized in critically ill patients, central venous catheters externally coated with 5-FU were found to be a safe and effective alternative to catheters externally coated with chlorhexidine and silver sulfadiazine. Another study found that azithromycin, which decreases QS-regulated virulence in *P. aeruginosa*, could help patients prevent ventilator-associated pneumonia (VAP). The findings suggest that virulence inhibition is a potential antimicrobial strategy due to azithromycin dramatically reducing the high risk of rhamnolipid-dependent VAP [116].

This innovative nonantibiotic therapy, which can suppress the expression of genes related to bacterial pathogenesis, prevent infection, and lessen the possibility of drug resistance in bacterial cells, has been gaining popularity in recent years. Recent studies have discovered numerous anti-QS compounds that can be used to regulate the pathogenic phenotypes of the majority of bacteria and to lessen the pathological harm in a variety of animal infection models [115,117,118,119]. However, anti-QS compounds may be toxic, and their therapeutic impact is not as permanent as that of antibiotics, limiting their widespread adoption. Combining anti-QS medicines with traditional antibiotics can considerably boost therapeutic medication efficacy. In contrast, the development calls for a mechanistic understanding of the QS system’s operation as well as an understanding of its molecular pathways to be used as the main application technique of anti-QS drugs for the treatment of bacterial diseases in the future. 

## 5. Conclusions

The global health problem caused by the introduction of several drug-resistant strains of ESKAPE nosocomial pathogens is extremely concerning. The QS signaling among these pathogens accompanies and elicits antimicrobial susceptibility and the production of bacterial virulence factors. Understanding the QS mechanism in ESKAPE pathogens opens the door to the creation of efficient QS-targeted drugs as substitutes for the drawbacks of traditional therapeutic choices. To prevent future bacterial pathogeneses and the antibiotic resistance caused by these difficult-to-treat pathogens, the complete QS pathways should be blocked or terminated.

## Figures and Tables

**Table 1:** Anti-QS strategies capable of bacterial virulence and antimicrobial resistance.

Strategies	Anti-QS Agents	Modes of Action	Effect on ESKAPE Organisms	References
Inhibition of AI synthases	Sinefungin	Structural analogues of *S*-adenosyl methionine (SAM) and acyl-carrier protein (ACP), the substrates of AHL synthases	Prevent bacterial infection and diminish QS-mediated virulence factors by blocking *P. aeruginosa* AHL synthesis	[64,65]
	Butyryl-SAM			
	L/D-S-adenosylhomocysteine			
	Triclosan	Reduction of the establishment of enoyl-ACP reductase precursors (FabI)	Decrease *S. aureus* AHL production	[66]
Targeting of QS Ligands	AHL lactonases (such as SsoPox, lactonase Aii810, QQ lactonase enzyme AHL-1, a novel lactonase cloned by bpiB01 and bpiB04 and lactonase AiiK)	Hydrolysis of the AHL lactone ring to form the consequent *N*-acyl homoserine	Lessen the extracellular proteases and pyocyanin biosynthesis, rhamnolipids, swarming motility and biofilm production and prevent bacterial infection of *P. aeruginosa*	[67,68,69,70,71,72]
	Acylases (i.e., *N*-acyl homoserine lactone acylase PA2385, acylase (EC.3.5.1.14)	Degradation of the AHL amide bond and generation of the corresponding free fatty acid and a lactone ring	Decrease elastase, pyocyanin synthesis and biofilm biomass and formation in *P. aeruginosa*	[73,74,75]
	Oxidoreductases (e.g., BpiB09)	Oxidation and consequent inhibition of signal QS molecules	Slow down bacterial motility and reduce biofilm formation and pyocyanin production in *P. aeruginosa*; hinder bacterial biofilm development and decrease the growth rate of *K. pneumoniae*	[76,77]
	3-Hydroxy-2-methyl-4(1H)-quinolone 2, 4-dioxygenase	Catalysis of the conversion of PQS to *N*-octanoylanthranilic acid and carbon monoxide	Hamper lectin A, pyocyanin and rhamnolipid biosynthesis of *P. aeruginosa*	[78]
	Quorum quenching antibody, RS2-1G9	Hydrolysis of 3-oxo-C12-HSL	Inhibit the activation of the mitogen- activated protein kinase p38 and protect against the cytotoxic effects of *P. aeruginosa* on macrophages generated from murine bone marrow	[79]
	XYD-11G2 antibody	Hydrolysis of 3-oxo-C12-HSL	Conquest the bacterial QS signals of *P. aeruginosa*	[80]
	Human single-chain variable fragments	Hydrolysis of 3-oxo-C12-HSL	Prevent mammalian cell apoptosis triggered by *P. aeruginosa*	[81]
	AP4-24H11 antibody	Targeting of autoinducing peptide-4	Induce the protective properties of *S. aureus*-produced abscess *in vivo*	[82]
	Synthetic RIP	Targeting of autoinducing peptide-4	Diminish *S. aureus* infections *in vivo*	[83]
Blockade of QS Transcriptional Regulators	Flavonoids	Allosteric blockade of the AI-binding receptors LasR and RhlR	Modulate the transcription of QS-controlled target promoters and limit the synthesis of virulence factors in *P. aeruginosa*	[84]
	*N*-decanoyl-L-homoserine benzyl ester	Repression of the quorum sensing control repressor	Impair the production of biofilms, swarming activity, and protease and elastase enzymes in *P. aeruginosa*	[85]
	Meta-bromo-thiolactone	Allosteric blockade of the AI-binding receptors LasR and RhlR	Decrease pyocyanin synthesis and biofilm development in *P. aeruginosa*	[86]
	A4, 4-bromophenyl-PHL B7, 4-iodo PHL C10 and 3-nitro PHL C14	Blockade of the AI-binding receptors, including TraR, LasR, and LuxR	Suppress the development of virulence factors in *P. aeruginosa*	[87]
	Virstatin	Inhibition of the expression of the *anoR* gene	Prevent bacterial movement and biofilm formation in *A. baumannii*	[88]
Probiotics	*L. plantarum* PA 100	Blockade of the function and inhibition of the synthesis of acyl homoserine lactones	Diminish biofilm production and elastase activity in *P. aeruginosa*	[89]
	*C. crustorum* ZHG 2-1	Degradation of C4-HSL and 3-oxo-C12-HSL	Suppress virulence factors (chitinase and protease), reduce swarming and swimming motilities, and inhibit biofilm formation in *P. aeruginosa*	[90]
	Cell-free acidic supernatants *L. lactis* NCDC 309, *L. rhamnosus* MTCC 5897, *L. rhamnosus* MTCC 5857, *L. fermentum* MTCC 5898, *L. acidophilus* NCDC 15, *L. delbrueckii* subsp. *lactis*, *L. plantarum* NCDC 372	Destruction of C4-HSL and 3-oxo-C12-HSL	Inhibit biofilm formation, elastase, and expression of *las*I and *rhl*I in *P. aeruginosa*	[91]
	*L. reuteri* RC-14	Inhibition of *arg* gene expression by bioactive cyclic dipeptides (known as 2,5-diketo-piperazines, or DKPs)	Neutralise *S. aureus* MN8 toxin TSST-1 synthesis (toxic shock syndrome)	[92]
	*L. plantarum*, *P. acidilactici*	Downregulation of genes including *cid*A, *ica*A, *dlt*B, *agr*A, *sortase*A, and *sar*A	Suppress the formation of *S. aureus* biofilm	[93]
Plant extracts	Eugenol	Suppression the expression of *las* and *pqs* systems	Prevent biofilm formation of *P. aeruginosa* PAO1.	[94,95,96,97]
		Reduction in the level of QS synthase genes, including *las*I, *rhl*I, and *rhl*A,	Inhibit biofilm growth and regressed virulence production (including pyocyanin, pyocyanin, and elastase) of *P. aeruginosa* PAO1	[98]
		Unknown	Limit the production of protease and pigments in MRSA	[99]
	Carvacrol (2-methyl-5-(1-methylethyl)-phenol)	Blocking *las*I and *las*R expression	Lower the biofilm development and bacterial motility of *P. aeruginosa*	[100,101]
	Phytol	Unknown	Suppress flagella mobilization, restricts the formation of pyocyanin, and inhibits the establishment of the biofilm in *P. aeruginosa* PAO1	[102,103]
	Sesquiterpene lactones	Unknown	Diminish the QS mediators in *P. aeruginosa* ATCC 27853	[104]
	Oleanolic aldehyde coumarate	Downregulation of *las*I*/las*R, *rhl*I/*rhl*R, and *gac*A expression	Decrease the *P. aeruginosa* ‘s biofilm biogenesis	[105]
	Linalool	Unknown	Prevent the establishment of *A. baumannii*‘s biofilms and alter this strain’s surface adhesion.	[106,107]

## Data Availability

Not applicable.
